# Clinical Experience and Recent Advances in the Development of *Listeria*-Based Tumor Immunotherapies

**DOI:** 10.3389/fimmu.2021.642316

**Published:** 2021-04-14

**Authors:** Mariam Oladejo, Yvonne Paterson, Laurence M. Wood

**Affiliations:** ^1^ Immunotherapeutics and Biotechnology, Texas Tech University Health Sciences Center, Abilene, TX, United States; ^2^ Microbiology, Perelman School of Medicine, University of Pennsylvania, Philadelphia, PA, United States

**Keywords:** *Listeria monocytogenes*, tumor immunotherapy, cancer vaccines, tumor antigens, vaccine vectors, clinical trials

## Abstract

The promise of tumor immunotherapy to significantly improve survival in patients who are refractory to long-standing therapies, such as chemotherapy and radiation, is now being realized. While immune checkpoint inhibitors that target PD-1 and CTLA-4 are leading the charge in clinical efficacy, there are a number of other promising tumor immunotherapies in advanced development such as *Listeria*-based vaccines. Due to its unique life cycle and ability to induce robust CTL responses, attenuated strains of *Listeria monocytogenes* (*Lm*) have been utilized as vaccine vectors targeting both infectious disease and cancer. In fact, preclinical studies in a multitude of cancer types have found *Listeria*-based vaccines to be highly effective at activating anti-tumor immunity and eradicating tumors. Several clinical trials have now recently reported their results, demonstrating promising efficacy against some cancers, and unique challenges. Development of the *Lm*-based immunotherapies continues with discovery of improved methods of attenuation, novel uses, and more effective combinatorial regimens. In this review, we provide a brief background of *Listeria monocytogenes* as a vaccine vector, discuss recent clinical experience with *Listeria*-based immunotherapies, and detail the advancements in development of improved *Listeria*-based vaccine platforms and in their utilization.

## Introduction

Tumor immunotherapy has gained rapid acceptance as an effective therapeutic strategy in the treatment of numerous malignancies. A multitude of methods to boost the anti-tumor immune response have been utilized including administration of purified immune system components to the use of microorganisms, such as attenuated bacteria and oncolytic viral particles, and have emerged as tools in the fight against cancer. While therapeutics that unleash the anti-tumor response by blocking inhibitory signaling pathways such as PD-1/PD-L1 have demonstrated the promise of tumor immunotherapy, immunotherapies that stimulate a specific anti-tumor response such as Sipuleucel-T, the first FDA-approved therapeutic cancer vaccine (1), have also provided a proof of concept for that approach. In fact, numerous cancer vaccines are currently in different stages of clinical trials with promising results (2).

Due to the challenge of overcoming tolerance within the tumor microenvironment, considerable effort has been directed towards stimulating the immune system to mount a robust response against these cells by targeting tumor-associated antigens (TAAs) and tumor-specific antigens (TSAs) (2). Necessary characteristics of an effective cancer vaccine are proficient antigen delivery, limited impact on normal healthy tissue, and the ability to elicit a robust anti-tumor immune response. In this regard, *Listeria monocytogenes*, a facultative gram-positive bacterium is an attractive platform for development of cancer vaccines due its ability to activate and deliver tumor antigens selectively to antigen-presenting cells, resulting in a robust anti-tumor cell-mediated immune response (3). It is these attributes of *Lm* that has driven significant development, in both academia and industry, of *Lm*-based tumor immunotherapies with several candidates in various stages of clinical development (**Table 1**). Therefore, in this review, our focus will be on the use of *Lm* as a tumor immunotherapy vaccine platform including a review of recent advancements in construction of improved *Lm*-based tumor immunotherapies, efficacy of *Lm*-based tumor immunotherapies in combination with other anti-cancer treatment modalities, and the current clinical experience with *Lm*-based tumor immunotherapies.

**Table 1:** Clinical Trials for *LC*-based Vaccines in Tumor Immunotherapy.

Trial Status	*Lm*-based Vaccine	Targeted Antigen(s)	Disease	Trial Phase	Enrollment	NCT Number*
**Active**	ADXS-NEO	Multiple personalized antigens	Multiple Cancers	Phase 1	5	NCT03265080
	ADXS-HPV	HPV16 E7	Cervical Cancer	Phase 3	450	NCT02853604
	ADXS-PSA	PSA	Prostate Cancer	Phase 1/2	51	NCT02325557
	ADXS-HPV	HPV16 E7	Cervical, Head and Neck Cancer	Phase 1/2	66	NCT02291055
	ADXS-HPV	HPV16 E7	Oropharyngeal Cancer	Phase 2	15	NCT02002182
	ADXS-HOT LUNG	Multiple antigens (hotspot mutations)	Non-Small-Cell Lung Cancer	Phase 1/2	74	NCT03847519
	CRS-207	Mesothelin	Pancreatic Cancer	Phase 2	63	NCT03190265
	CRS-207	Mesothelin	Pancreatic Cancer	Phase 2	70	NCT03006302
**Completed,** **Withdrawn, Terminated**	ADXS31-164	Her2	HER2-Expressing Solid Tumors	Phase 1/2	12	NCT02386501
	ADXS-HPV	HPV16 E7	Anal, Rectal Cancer	Phase 2	51	NCT02399813
	ADXS-HPV	HPV16 E7	Anal Cancer	Phase 1/2	11	NCT01671488
	ADXS-HPV	HPV16 E7	Cervical Cancer	Phase 1/2	25	NCT02164461
	ADXS-HPV	HPV16 E7	Cervical Cancer	Phase 2	54	NCT01266460
	CRS-207	Mesothelin	Pancreatic Cancer	Phase 2	303	NCT02004262
	ADU-623	EGFRvIII, NY-ESO-1	Astrocytic Tumors, Glioblastoma Multiforme, Anaplastic Astrocytoma, Brain Tumor	Phase 1	11	NCT01967758
	ANZ-100 (CRS-100)	N/A	Carcinoma and Liver Metastases	Phase 1	9	NCT00327652
	JNJ-64041809	Multiple prostate antigens	Prostate Cancer	Phase 1	26	NCT02625857
	ADXS-HPV	HPV16 E7	Cervical Intraepithelial Neoplasia	Phase 2	81	NCT01116245
	ADXS-HPV	HPV16 E7	Oropharyngeal Carcinoma	Phase 1	2	NCT01598792
	pLADD	Multiple personalized antigens	Colorectal Cancer	Phase 1	28	NCT03189030
	CRS-207	Mesothelin	Malignant Pleural Mesothelioma	Phase 1	60	NCT01675765
	CRS-207	Mesothelin	Malignant Pleural Mesothelioma	Phase 2	10	NCT03175172
	CRS-207	Mesothelin	Pancreatic Cancer	Phase 2	93	NCT02243371
	CRS-207	Mesothelin	Gastric, Gastroesophageal Junction, Esophageal Cancers	Phase 2	5	NCT03122548
	CRS-207	Mesothelin	Malignant Epithelial Mesothelioma, Pancreatic, Ovarian, Non-Small-Cell Lung Cancer	Phase 1	17	NCT00585845
	CRS-207	Mesothelin	Ovarian, Fallopian, Peritoneal Cancer	Phase 1/2	35	NCT02575807
	CRS-207	Mesothelin	Pancreatic Cancer	Phase 2	93	NCT01417000
	JNJ-64041757	EGFRvIII, Mesothelin	Non-Small-Cell Lung Cancer	Phase 1	18	NCT02592967
	JNJ-64041757	EGFRvIII, Mesothelin	Lung Cancer	Phase 1/2	12	NCT03371381
	ADXS-HPV	HPV16 E7	Non-Small-Cell Lung Cancer	Phase 2	124	NCT02531854
	JNJ-64041809	Multiple prostate antigens	Prostate Cancer	Phase 2	0	NCT02906605

*Additional clinical trial information for each study available at https://clinicaltrials.gov/ct2/home

## Development of Inactivated and Attenuated Bacteria as Tumor Immunotherapeutics

Evidence that bacteria can harness the immune system to elicit therapeutic efficacy against malignancies dates to the early twentieth century. While William Coley laid a solid foundation for modern bacterial immunotherapy, there are other notable scientists whose work predated even his discoveries. In 1851, Belgian surgeon Didot pioneered the use of a syphilis vaccination to treat inoperable cancer (4). In 1868, Busch, a German scientist, reported the efficacy of erysipelas infection in treatment of sarcoma in a crudely performed clinical study (5–7). Two decades later in 1882, Friedrich Fehleisen discovered *Streptococcus pyogenes* to be the causative agent of erysipelas and noticed that infection with this bacterium caused transplanted tumors to melt away (5, 6, 8). It was armed with these observations and the serendipitous recovery of a German immigrant with an inoperable neck sarcoma, that William Coley embarked on the challenging but foundational work of tumor immunology (9). His belief that a component or “factor” from the microbes, rather than the whole live microorganism, is responsible for the oncolytic activity he observed led to the creation of his Coley toxins, a mixture of heat killed *Streptococcus pyogenes* and *Serratia mersacems* (9). In fact, this novel therapy was reported to result in partial or complete remissions in many difficult-to-treat patients (10, 11). However, given our limited understanding of immunology at the time, the mechanisms that would explain the efficacy of this strategy were unclear, thereby limiting the continued development of Coley’s toxins. Eventually, the advent of radiotherapy and chemotherapy that could be more easily standardized relegated this novel therapeutic strategy, comprised of killed or inactivated bacteria, to the background. As our understanding of the immune system has matured, there has been renewed interest in the use of attenuated, inactivated, and killed bacteria to stimulate anti-tumor immunity.

## Use of *Listeria Monocytogenes* as a Tumor Immunotherapeutic to Elicit Tumor Specific Immunity

Unlike the approach taken by Coley, whose toxins non-specifically resulted in an anti-tumor response, current approaches focus on achieving complete and durable antitumor immunity through induction of tumor-specific immune responses. To elicit this tumor-specific immunity, the strategy employed by most tumor immunotherapies is to activate cytotoxic T lymphocytes (CTLs) that recognize peptides from tumor-specific and/or tumor-associated antigens presented on MHC Class I and target tumor cells for lysis (12). In fact, CTL-mediated tumor cell destruction occurs commonly in a process called immunosurveillance, in which the immune system recognizes and eliminates malignant cells prior to clinical detection (13). Unfortunately, some malignant cells can escape immune-mediated destruction and develop into a clinically relevant tumor. The tumors that have escaped immunosurveillance have been able to achieve this, in part, by fostering the development of an immunosuppressive microenvironment that impedes the function and survival of responding lymphocytes, including cytotoxic T lymphocytes. In order to overcome this immunosuppressive microenvironment, various strategies have been employed to enhance the activation and mobilization of CTLs in the tumor environment such as the use of oncolytic viruses, dendritic cell vaccines, adoptive cell therapy, and microbial vectors for the targeting of TAAs (14–16). Each of these strategies has their own advantages and disadvantages. Aside from the important consideration of toxicity in the use of oncolytic viruses, off target viral replication and development of neutralizing antibodies may prevent recurrent use of this strategy. Bacterial vectors such as *Streptococcus pyogenes*, *Clostridium novyi*, *Salmonella enterica*, and *Listeria monocytogenes* (17–20), are all vectors that have been used in cancer immunotherapy and do not pose this significant drawback of preexisting neutralizing immunity. However, what differentiates *Listeria monocytogenes* from its vaccine peers and makes it a superior vector for delivery of cancer antigens is its unique life cycle. *Lm* is readily taken up by macrophages in the course of an infection and, once within a phagocytic compartment, expresses and secretes a cytolysin, Listeriolysin O (LLO) (21). LLO, along with bacterial phospholipases, disrupts the integrity of the phagosome and allows *Lm* to escape into the cytosol and elude destruction in the phagolysosome (22). Once in the cytosol, *Lm* proliferates and secretes additional virulence factors that propel it within the cell and into adjacent cells in order to propagate the infection (23). It is this life cycle that makes *Lm* an ideal candidate to deliver antigen to both the MHC I and II pathways, activating CD4^+^ T cells and, most importantly for tumor-lytic responses, cytotoxic CD8^+^ T cells (24). In fact, infection with *Lm* elicits a robust and long-lasting immunological memory response that provides protection against infection upon future exposure to the pathogen (25–27).

**Figure 1:**
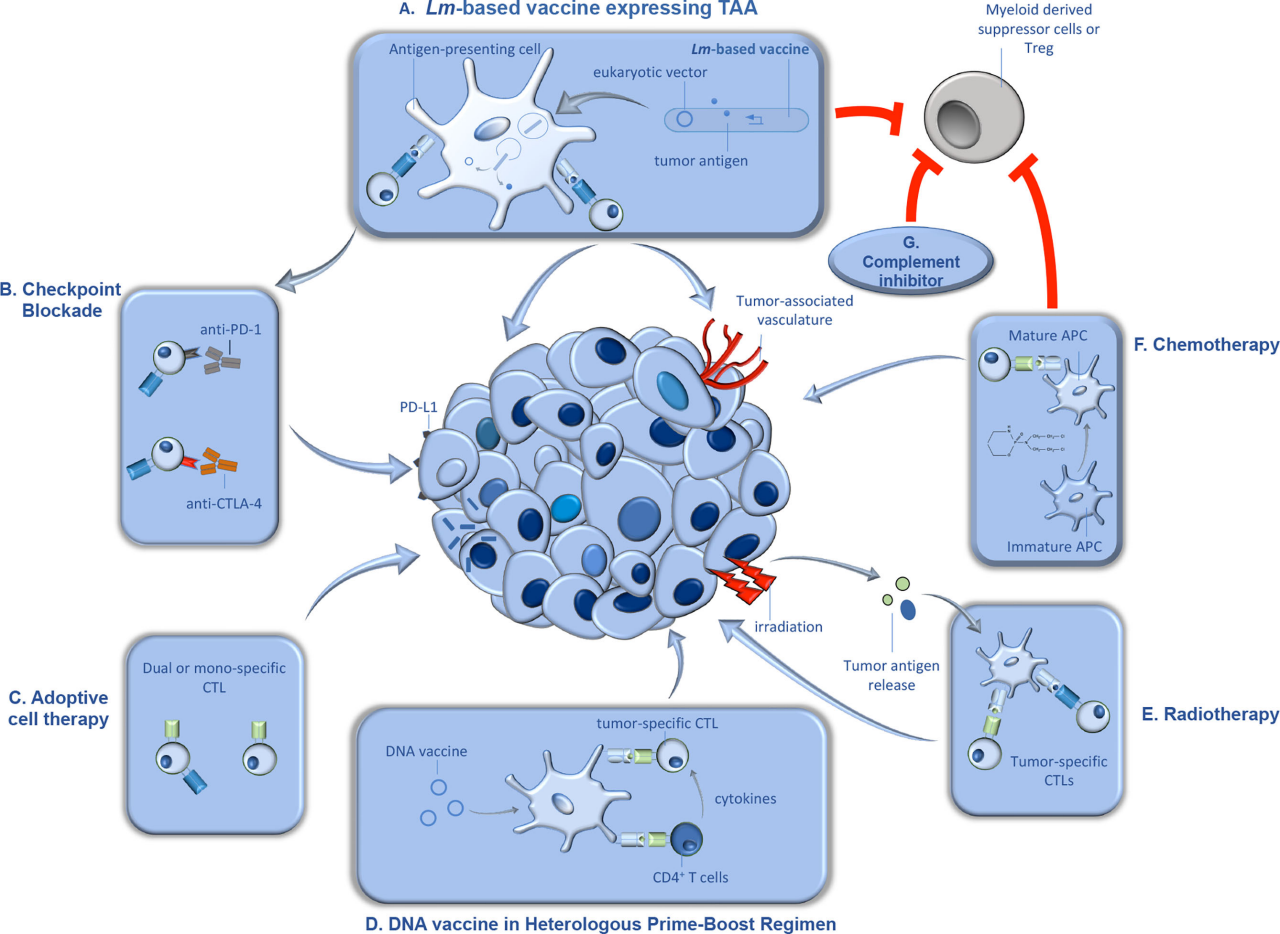
Synergistic therapeutic approaches with *Lm*-based vaccines in tumor immunotherapy. *Lm*-based vaccines have found therapeutic success in preclinical models of cancer for decades, and recent studies demonstrated significant promise for this type of active tumor immunotherapy in clinical trials. Further, recent studies suggest that the anti-tumor efficacy of *Lm*-based vaccines can be significantly improved when utilized in combination with synergistic anti-cancer therapeutics. In this figure, we detail some of the anti-cancer therapeutics that demonstrated efficacy in combination *Lm*-based vaccines along with their proposed mechanism(s) of action. **(A)** Upon administration, attenuated *Lm* vaccines infect antigen presenting cells in secondary lymphoid organs and gain entry to the cytosol, wherein they produce and secrete tumor antigen and/or release eukaryotic expression vectors encoding for tumor antigen. Once delivered by *Lm*, the tumor antigens go through processing and presentation to naïve tumor-specific CD4^+^ and CD8^+^ cytotoxic T lymphocytes (CTLs). Tumor-specific CTLs, activated through the action of *Lm*-based vaccines, migrate to the tumor microenvironment (TME) and lyse tumor cells and/or cells associated with the tumor vasculature. Importantly, *Lm*-based vaccines can also reduce immunosuppression within the TME by reducing tumor-associated MDSCs and Tregs. **(B)** Upon continuous activation, tumor-specific CTLs can become exhausted, characterized by the upregulation of inhibitory molecules such as programmed cell death protein 1 (PD-1) and cytotoxic T-lymphocyte-associated protein 4 (CTLA-4). Antibody-mediated blockade of inhibitory molecules results in enhanced T cell function and synergy with *Lm*-based vaccine anti-tumor responses. **(C)** Dual or mono-specific adoptively transferred CTLs targeting both tumor antigens and/or *Lm*-derived antigens work in concert with *Lm-*based vaccines, delivered intratumorally, through increasing the breadth of the anti-tumor T cell response. **(D)** *Lm*-based vaccines have also been found to be effective in heterologous prime-boost approaches. DNA-based tumor vaccines, used as either prime or boost in a heterologous prime-boost vaccination schedule with *Lm*-based vaccines, induce robust expansion of Th1 helper T cells that produce cytokines in support of responses by tumor-specific CTLs. **(E)** Further, radiation can synergize with *Lm*-based vaccines, in part, by causing direct tumor death, resulting in release of tumor antigens which are processed and presented by APCs to tumor-specific T cells. **(F)** Chemotherapeutic agents such as cyclophosphamide can also synergize with *Lm*-based vaccines, in part, by facilitating maturation of APCs. However, cyclophosphamide can also reduce immunosuppressive cell types, Tregs and MDSCs, in the TME allowing for improved anti-tumor efficacy by the anti-tumor responses induced by *Lm*-based vaccines. **(G)** Similarly, inhibition of complement signaling can synergize with *Lm*-based vaccines, in part, through limiting recruitment of MDSCs and Tregs to the TME.

In addition to its unique life cycle, *Lm* affords a number of advantages as a vector for tumor immunotherapy. Previous studies found that antigens encoded by *Lm* constructs are more efficiently delivered to the protein processing and presentation machinery than those encoded by other bacterial vectors (28). Further, *Lm* vectors have the ability to break immunologic tolerance, *via* reduction of immunosuppressive myeloid-derived suppressor cells (MDSCs) and regulatory T cells (Tregs) within the tumor microenvironment. Finally, in addition to selective uptake in the spleen and liver of infected subjects, *Lm* displays a specific tropism for primary and metastatic tumors (29, 30). These attractive features, including the ease of manipulation and attenuation of this organism, have been harnessed by various groups in development of attenuated strains of *Lm*, expressing a wide variety of tumor antigens. We have extensively discussed the various ways by which these attenuated strains are generated, and the antigens that have been delivered by this therapeutic platform (31). Described below, are the more recent *Lm* construction trends as well as the use of these various constructs in combination therapy depicted in **Figure 1** that are currently being tested in various preclinical and clinical trials (**Figure 1**).

## Clinical Experience With *Lm*-Based Vaccines for Cancer

With the clinical experience of *Lm*-based vaccines in oncology going well beyond a decade, the promise and further challenges are now being realized regarding their place in the future oncologists toolkit. Below, we summarize clinical studies that have recently published their findings for an array of cancers. Importantly, these studies only scratch the surface of clinical studies regarding the efficacy of *Lm*-based vaccines for cancer that are currently underway or not yet peer-reviewed and published as summarized in **Table 1**.

### HPV-Associated Cancers

As the development of *Lm*-based vaccine vectors matured in the late 1990’s, the focus of constructing novel vectors progressed from those that targeted model tumor antigens, in order to better understand the platform, to the incorporation of clinically relevant tumor antigens. One of the first *Lm*-based vaccines to incorporate a clinically relevant tumor antigen targeted Human papillomavirus (HPV)-associated cancers (3). While most individuals clear infections with HPV, in certain individuals that are unable to clear the infection, chronic infection with high-risk strains of HPV, such as HPV 16 and 18, can increase their risk of developing cancer (32). Infection with high-risk HPV is particularly problematic in women as it can result in carcinoma of the cervix (33). However, high-risk strains of HPV are also associated with head and neck cancers among others (34). In HPV-associated cancers, constitutive expression of the HPV oncoproteins, E6 and E7, is required to maintain the malignant phenotype (35). Therefore, this necessary expression by the tumor cells, and the lack of central tolerance to these viral antigens, make these oncoproteins particularly attractive targets for *Lm*-based tumor immunotherapy.

In the initial preclinical study documenting the development of *Lm*-based vaccines targeting HPV-associated cancers, there were two vaccines constructed, *Lm*-E7 and *Lm*-LLO-E7 (3). While *Lm*-E7 expressed and secreted HPV 16 E7, generated E7-specific CTLs, and demonstrated anti-tumor efficacy, this was insufficient to cure a majority of mice with established tumors expressing E7. However, administration with an *Lm* construct that expressed and secreted HPV 16 E7 genetically fused to a truncated non-hemolytic form of Listeriolysin O (tLLO) did result in complete eradication of E7-expressing tumors in all experimental mice. The adjuvant-like properties of tLLO have since been elucidated and the vast majority of *Lm*-based vaccines in clinical and preclinical testing genetically fuse the tumor antigen to either tLLO or a truncated form of another *Lm*-derived protein with adjuvant-like properties, ActA (36–39).

The first clinical use of *Lm* -LLO-E7 (also known as ADXS-HPV and as ADXS11-001), or any *Lm*-based therapy in cancer patients, was a Phase I clinical trial in women with cervical cancer that had failed previous front-line therapy including surgery, chemotherapy, and radiation (40). The patients were enrolled into three groups of 5 with each group receiving two intravenous doses ranging from 10^9^ colony-forming units (CFU) of *Lm*-LLO-E7 to 10^10^ CFU given 22 days apart. As would be expected with intravenous (i.v.) administration of *Lm*, all of the patients experienced adverse events (AE) including fever, chills, nausea, and vomiting with 40% experiencing severe grade 3 AEs but none of the patients experienced a life-threatening grade 4 adverse event (AE) due to the treatment. Dose-limiting toxicities occurred in the group receiving the highest dose with one patient requiring antibiotics to mitigate a fever. In terms of efficacy, nearly half of the patients were reported to have stable disease with one receiving a possible partial response and the median overall survival was 347 days. These results warranted further investigation as they demonstrated that *Lm*-based vaccines have a safety profile that is likely more favorable than salvage chemotherapy and efficacy that may improve upon current treatment modalities.

Following the promising safety profile of *Lm*-LLO-E7 observed in the Phase I clinical trial, two Phase II trials were commenced to further characterize its safety and efficacy in patients with recurrent cervical cancer. In the first trial, 109 patients were separated into two groups with one receiving only 10^9^ CFU *Lm*-LLO-E7 and the second group receiving *Lm*-LLO-E7 along with cisplatin (41). While the *Lm*-LLO-E7 group received three i.v. administrations each separated by roughly one month, the *Lm*-LLO-E7 + cisplatin group received their initial dose of *Lm*-LLO-E7 and then waited 4 weeks to receive their 5 weekly doses of cisplatin and finally the second dose of *Lm*-LLO-E7. The rationale for this staggered administration of *Lm*-LLO-E7 and cisplatin was to promote treatment synergy while limiting any possible suppressive effect of cisplatin on the immune response to *Lm*-LLO-E7. However, the addition of cisplatin to *Lm*-LLO-E7 did not have a significant impact on efficacy. The overall response rates (ORR) were comparable between the groups with an ORR of 17.1% for the *Lm*-LLO-E7 group and 14.7% for the *Lm*-LLO-E7 +cisplatin group. Similarly, median overall survival (OS) were not statistically significant with 8.28 and 8.78 months in the *Lm*-LLO-E7 and *Lm*-LLO-E7+ cisplatin groups, respectively. In terms of safety, the difference between these two arms was more pronounced with more AEs observed in the *Lm*-LLO-E7+cisplatin group (429) than the *Lm*-LLO-E7 alone group (275) but most of these AEs were mild to moderate in severity (80.4%). These results informed the second Phase II trial for cervical cancer funded by the NCI, wherein, there was only a single arm receiving three doses of *Lm*-LLO-E7 only in the first stage, with some patients receiving an additional three doses in a second stage (42). Treatment-related AE types and frequencies were similar to previous trials with nearly all patients experiencing at least one and 38% experiencing a grade 3 AE. In addition, 4% of patients (2) experienced a grade 4 AE that consisted of sepsis and cytokine release syndrome. In terms of efficacy, the median OS was 6.1 months and the 12-month OS rate was 38% which met the study goal of 35%, a dramatic improvement on the historical 12-month OS of 21% in a similar patient population. This trial along with other ADXS-HPV trials were placed on temporary hold when evidence of listeriosis was observed in a patient 31 months after the last administration. This was believed to be due to persistence associated with biofilm formation on prosthetic material at recent fracture sites in the patient (43). These Phase II trials demonstrated that *Lm*-LLO-E7 is tolerated relatively well in comparison to other treatment modalities and has significant promise to extend the lifespan of a poorly treated patient population.

The results from the Phase I and Phase II clinical trials of *Lm*-LLO-E7 in cervical cancer resulted in the commencement of a Phase III clinical trial, AIM2CERV (NCT02853604). While AIM2CERV is still ongoing and results have not yet been published, the recruitment of new patients has been halted by its sponsor, Advaxis Inc., in order to allocate resources to the development of other promising upcoming vaccine platforms. However, clinical trials are still ongoing with *Lm*-LLO-E7 to evaluate the efficacy of *Lm*-LLO-E7 in other HPV-associated cancers, but the halt of AIM2CERV suggests it may take longer than previously anticipated before possible FDA approval and wide availability of *Lm*-LLO-E7.

In addition to cervical cancer, a recent study reported promising results with *Lm*-LLO-E7 in another HPV-associated cancer, anal cancer (44). In this Phase 1/2 clinical trial, patients with non-metastatic anal cancer were administered *Lm*-LLO-E7 once before and three times after receiving chemoradiation consisting of mitomycin C, 5-fluorouracil, and daily image-guided radiation therapy. While most patients experienced only low-grade AEs due to the *Lm*-based vaccine, two patients did experience grade 3 chills/rigor but all AEs were managed and resolved within 24 hrs. The clinical response was also highly promising as all 9 patients experienced complete responses by trial end (42 months) with only one patient progressing to metastatic disease six months post-trial. This study suggests *Lm*-LLO-E7 may have a promising future as part of a front-line therapeutic regimen in the treatment of HPV-associated cancers beyond cervical cancer.

### Pancreatic Cancer

Pancreatic ductal cell carcinoma (PDAC) is one of the most challenging malignancies to treat with the most effective current treatments being surgery and chemotherapy (45). Unfortunately, while the advancement of immunotherapy in oncology, particularly immune checkpoint inhibition (ICI), has brought about significant survival benefits to many cancer patients, PDAC patients have not been afforded the same benefit (46). PDAC expresses TAAs such as mesothelin but is poorly infiltrated by T cells and is considered an immunologically “cold” tumor (47–49). As *Lm*-based vaccines have been found to modulate the tumor microenvironment (TME) and make it more amenable to anti-tumor immune responses (50), significant effort has been expended to determine the promise of this vaccine platform in the treatment of PDAC. To target mesothelin-expressing PDAC, an *Lm*-based vaccine was constructed that expressed and secreted human mesothelin genetically fused to the first 100 residues of ActA, ActAN100, in a highly attenuated strain of *Lm*, LADD (51). This mesothelin-targeting *Lm*-based vaccine (*Lm*-Mesothelin, CRS-207) was initially brought into the clinic in a Phase I trial with several cancer types that all express mesothelin, including PDAC (52). Study subjects received at least one i.v. administration of CRS-207 at a particular dose ranging from 10^8^ to 10^10^ CFUs. The vaccine was generally well tolerated with the majority of patients experiencing grade 2 or less AEs. However, a number of patients experienced grade 3 AEs and at least 2 grade 4 AEs at the highest doses. Therefore, the maximum tolerated dose was determined to be 10^9^ CFUs. While the study was not powered to assess efficacy, 37% of patients survived beyond 15 months. Interestingly, the patients with longer survival had a more robust T cell response to the vaccine.

Due to the promising data from the Phase I clinical trial, three separate Phase II clinical trials were commenced to evaluate the efficacy and safety of CRS-207 in PDAC. The first Phase IIa trial with 90 patients assessed the efficacy of cyclophosphamide (Cy), a chemotherapeutic drug that limits peripheral tolerance, in combination with GVAX, a tumor vaccine consisting of irradiated allogeneic PDAC cells lines secreting GM-CSF (Arm B) or a heterologous prime boost regimen with Cy/GVAX with CRS-207 (Arm A) (53). The heterologous prime boost regimen, Cy/GVAX+CRS-207, was superior to the Cy/GVAX only treatment regimen, in terms of median OS (6.1 vs. 3.9 months) and in terms of 12-month survival (24% vs. 12%). In fact, mesothelin-specific CD8^+^ T cell responses were observed earlier with Cy/GVAX+CRS-207 than Cy/GVAX only suggesting that the expected synergy of the prime boost approach was realized in terms of both efficacy and anti-tumor immune response. The most common AEs were associated with injection site reactions but a majority of patients also experienced fever, chills, and gastrointestinal symptoms. The most prevalent severe AE (grade 3-4) was lymphopenia that was observed in 44% of patients. However, one patient did develop systemic listeriosis 12 days post administration that resolved after receiving i.v. penicillin (54). The promising results demonstrate the ability of an *Lm*-based vaccine to synergize with another vaccine strategy, in this case Cy/GVAX, in a heterologous prime boost regimen, thereby, providing strong rationale for the continued exploration of the ability of immunotherapy to increase survival in PDAC.

Unlike the first Phase IIa clinical trial with CRS-207, the second trial (ECLIPSE study) included groups to assess the therapeutic efficacy of CRS-207 by itself in comparison to standard-of-care chemotherapy and a CRS-207+Cy/GVAX combination treatment regimen (55). The primary group in this study was patients given each of these interventions as a third+ line therapy. In this population, the median OS of chemotherapy alone was 4.6 months vs. 3.7 months with the Cy/GVAX+CRS-207 regimen. Interestingly, the CRS-207 alone treatment group had a median OS of 5.4 months suggesting some promise as a therapy in PDAC but these differences in median OS were not significant between any of the treatment groups. In a smaller group of patients when each of these treatment regimens were given as a second line therapy, there was no significant difference in median OS but the chemotherapy treatment arm had the highest median OS. The lower median OS in this trial compared to the first Phase II trial with CRS-207 was likely due to the patients in this trial having more advanced disease. As with the previous CRS-207 studies, treatment related severe AEs were minimal with the majority of AEs being low-grade. This trial again demonstrates the challenges of demonstrating efficacy in PDAC and suggested that a different approach may be required to demonstrate the promise of *Lm*-based vaccines in this disease.

As the previous Phase II studies with CRS-207 suggested, a new therapeutic approach would be required to demonstrate that it is capable of improving PDAC patient survival. As ICI therapy has been found to synergize with *Lm*-based vaccine approaches preclinically, a third Phase II trial with CRS-207 was conducted in PDAC patients that included anti-hPD-1 blockading antibody, nivolumab, along with Cy/GVAX+CRS-207 (Arm A) in comparison to Cy/GVAX+CRS-207 alone (Arm B) (56). The median OS for each arm was similar at 5.9 and 6.1 months for Arm A and Arm B, respectively. While the addition of nivolumab did not appear to improve median OS in patients receiving Cy/GVAX+CRS-207, some other parameters such as disease control rate and 12- and 18-month survival were improved beyond Cy/GVAX+CRS-207 alone. Interestingly, in the patients receiving nivolumab, long-term survival was correlated with increased CD8^+^ T cell infiltration into tumors and a reduction in likely immunosuppressive tumor-associated myeloid cells. However, nivolumab treatment did result in more AEs in patients but these were as expected in ICI therapy with 2.2% of patients, all in Arm A, discontinuing treatment due to treatment-related AEs. This study again highlights the enormous challenges of improving survival in PDAC patients. While the Phase II clinical studies with CRS-207 in PDAC did not meet their primary efficacy endpoints, they have advanced our knowledge and experience with *Lm*-based vaccines in a particularly difficult-to-treat patient population that will hopefully inspire additional studies with improved treatment regimens.

### Malignant Pleural Mesothelioma

Malignant pleural mesothelioma is a rare disease caused by exposure to asbestos with a high mortality rate (57). The previous first line therapy for MPM was pemetrexed with cisplatin that afforded patients a median OS of 14.1 months, until the recent approval of nivolumab and ipilumimab that raised median OS to 18.1 months in the Checkmate-743 Phase III trial (58, 59). The clinical success of ICI therapy in MPM suggests that it is a disease that is receptive to immunotherapies such as *Lm*-based vaccines. As CRS-207 targets mesothelin, a tumor antigen highly expressed in MPM, a Phase I clinical trial was conducted to determine safety and tolerability of CRS-207 in combination with pemetrexed and cisplatin. In contrast to its efficacy on PDAC, 89% of patients receiving CRS-207 in combination with pemetrexed and cisplatin experienced tumor reduction (60). While the median OS was comparable to chemotherapy at 14.7 months, the OS rate at 1 year of 64.9% was more comparable to ICI-treated MPM patients. As a correlate to this promising efficacy, percentages of functional CD8^+^ T cells increased and immunosuppressive M2 macrophages decreased in tumors over the course of treatment. AEs were similar to previous trials with CRS-207 and there was no additive effect with the addition of chemotherapy. While only a Phase I study with less than 40 patients, the promising results provide a strong rationale for the continued assessment of CRS-207 in MPM and possibly in combination with the recently FDA-approved ICI therapeutics to determine any synergistic potential.

### Non-Small Cell Lung Cancer

Lung cancer is responsible for more deaths worldwide than any other form of cancer with non-small cell lung cancer (NSCLC) accounting for the vast majority (61–63). Effective therapeutic options are lacking for NSCLC as even ICI therapy provides relatively limited benefits compared to other cancers, with a 5-year survival of 15-25% (64, 65). Due to the previously reported ability of *Lm*-based immunotherapies to reduce immunosuppression in the TME and activate tumor-specific immunity, a Phase Ib/2 trial was commenced to determine the safety and effectiveness of *Lm*-based vaccination alone or *Lm*-based vaccination in combination with ICI in NSCLC (66). The bivalent *Lm*-based vaccine utilized in these trials, JNJ-75, expresses NSCLC-associated antigens, both EGFRvIII and mesothelin, on the LADD vector platform, and was previously developed by Aduro Biotech as ADU-214 (31). As a monotherapy, 18 patients received JNJ-75 administered twice i.v. in either 10^8^ or 10^9^ CFUs/dose. Each dosage of JNJ-75 was well tolerated with no dose-limiting toxicity, and the expected pyrexia and chills with duration no longer than 24hrs. Similar safety data was seen in the combination study where all 12 patients received 10^9^ CFUs of JNJ-75 along with 240mg nivolumab. In total, only 6 treatment-related serious AEs (grade 3 or higher) were observed and bacterial shedding was not found in blood, fecal, or urine samples at 4hrs, 2 days, and 4 days-post administration. In terms of clinical response, 44% of patients had stable disease as the best overall response while one patient had a partial response in a target lesion identified at the beginning of the trial but still had overall disease progression. In the combination trial, the best overall response was stable disease in 4 patients but the trial was stopped early and efficacy data was limited. In the monotherapy study, levels of serum proinflammatory cytokines were elevated and activation of T cells and NK cells was increased at 24hrs post administration with cytokine levels returning to baseline at 48hrs. Further, the magnitude of mesothelin-specific T cells responses was limited compared to recall responses to tetanus toxoid and influenza. While stable disease was observed in some patients and there was clear evidence of inflammatory responses, the overall lack of significant clinical benefit to patients suggests that JNJ-75 alone or in combination with nivolumab will not proceed to further with clinical development.

### Osteosarcoma

Osteosarcoma is a highly aggressive form of cancer that overwhelmingly affects children and primarily manifests in the long bones prior to metastasizing to vital organs (67). The standard of care currently consists of resection of the lesion or amputations of the affected limb and chemotherapy (68). However, many patients experience recurrence of the disease due to the seeding of micrometastases prior to primary tumor resection (69). Much like in breast cancer, a proportion of pediatric osteosarcoma patients have lesions that highly express the EGFR family receptor, HER2/neu, that correlates with poor prognosis (70). Importantly, a Phase Ib clinical trial has recently completed utilizing a human Her2/neu-targeting *Lm*-based vaccine, ADXS31-164, for adult patients with Her2/neu-expressing tumors (NCT02386501). This vaccine has now been licensed for development in the pediatric osteosarcoma setting by OS Therapies in conjunction with the NCI Children’s Oncology Group. While human clinical studies have not yet begun targeting human osteosarcoma with *Lm*-based immunotherapy, the promise of this therapeutic approach in humans may be predicted by the recently completed and published results from clinical trials in canines for osteosarcoma utilizing ADXS31-164. Canine osteosarcoma is highly aggressive much like the human disease in terms of prognosis and treatment (71). In fact, a significant proportion of canine osteosarcomas overexpress the tumor antigen Her2/neu much like their human counterparts (70, 72). Since human Her2 and canine Her2 have >90% homology and the *Lm*-based vaccine targeting human Her2/neu, ADXS31-164, had already been developed and successfully tested in mice, a small Phase I clinical trial consisting of 18 client-owned dogs was performed to determine the safety, tolerability, and efficacy of the *Lm*-based vaccine targeting Her2/neu, ADXS31-164, in canine osteosarcoma (73, 74). For this trial, dogs were treated with three doses of ADXS31-164 once every three weeks following standard of care amputation and carboplatin adjuvant chemotherapy. AEs were generally low-grade, transient, and independent of ADXS31-164 dosage. Strikingly, the 1, 2, and 3-year survival rates for ADXS31-164 treated dogs were 77.8%, 67%, and 56%, respectively. This is in stark contrast to dogs in a historical control group treated with standard of care amputation and adjuvant chemotherapy with 1, 2, and 3-year survival rates of 55%, 28%, and 22%, respectively. In addition to increased survival, Her2-specific T cell responses were also observed in 83% of the dogs in the study. Based on the promising results of this study, ADXS31-164 was granted conditional approval by the USDA and the study was continued and expanded. In fact, a subsequent study of ADXS31-164 consisting of 50 dogs with osteosarcoma was recently reported. The study protocol mimicked the early Phase I trial and the observed AEs generally confirmed the low-grade and transient nature of those reported in the first study (75). However, 4 dogs did develop listeriosis, a finding not observed in the initial trial. The severity of the listeriosis varied between the animals with some requiring antibiotic treatment. One animal presented with an extrapleural mass that cultured positive for ADXS31-164 three weeks after receiving the final vaccine dose, and required surgery to remove the chest abscess (76). Unfortunately, the study was unable to report efficacy data due to 30% of the dogs discontinuing treatment, an inability to complete follow-up evaluations, and concurrent or subsequent therapies after ADXS31-164 treatment that complicated analysis.

### Future of *Listeria* in the Clinic

Clinical trials continue for a number of *Lm*-based vaccines (**Table 1**). However, with the discontinuation of the AIM2CERV Phase III trial, it may take longer than previously anticipated before an *Lm*-based vaccine is widely available for use in oncology. While the safety profile of *Lm*-based vaccines has been very tolerable in comparison to chemotherapy and other forms of immunotherapy, the recent rare reports of listeriosis after final dosing in both humans and dogs may be a challenge in a small number of patients going forward (43, 76). As current trials already incorporate a regimen of antibiotics subsequent to dosing with *Lm*-based vaccines, this regimen may need to be modified and extended in order to mitigate the possible risk of listeriosis. Further, development of even more attenuated vaccine strains described below may help further mitigate this risk and, along with the combination of synergistic therapies, may spur greater clinical success for future *Lm*-based vaccines.

## New Trends in *Lm* Vaccine Constructs

For more than two decades, *Listeria monocytogenes* has proven itself to be a highly promising vector for tumor immunotherapy in numerous preclinical studies (31). While the currently available platforms have demonstrated promising performance in clinical trials, none has yet been approved for clinical use by the FDA, thus there is continued interest in engineering newer, safer and more effective *Listeria monocytogenes*-based vaccine platforms (77–79). In addition, while numerous clinical studies have confirmed an excellent safety profile for *Lm*-based vaccines in healthy individuals and oncology patients, there have been some concerns regarding the ability of current clinical strains to persist in patients (40, 43, 52, 66, 76, 80). Described below are the recent advances at overcoming these challenges and improving the safety and efficacy of *Lm*-based immunotherapies

### Enhanced Attenuation Methods

While infection with the virulent wild-type strain of *Listeria monocytogenes* can lead to the formation of robust memory T cell responses, several studies have found that attenuated strains of *Lm* actually result in improved immune memory and protective responses (81, 82). The methods utilized to attenuate *Lm* primarily revolve around deletion of non-essential virulence genes that allow for sufficient infectivity and antigen production but limit the potential for severe infection, a necessary concern when the patient population for tumor immunotherapy may already suffer from immune deficiency (83). In fact, the *Lm*-based vaccines currently or previously in clinical trials have all contained some form of deletion of virulence genes such as *actA* or *prfA (* 31). While the safety of these attenuated *Lm* platforms has been demonstrated in multiple trials, further modifications that enhance attenuation and improve therapeutic efficacy of *Lm*-based vaccines continue.

One such advancement is the development of suicidal strains that are programmed to lose viability once the antigenic cargo has been delivered to an antigen-presenting cell (84). The need for these suicidal vaccines stems from instances where, despite attenuation, persistence of *Lm* vaccine constructs has been observed following the administration of live *Lm* vaccines (43, 85). Early work in the development of a *Listeria* suicide strain utilized an *Lm* construct that produced a phage lysin under the control of the ActA promoter so that the suicide switch would be engaged once *Lm* gained entry to the cytosol of an APC and delivered its cargo (84). A similar approach by Souders et al., that we discuss further in 1.4.4, found a dramatic reduction in death of infected APCs by a suicidal strain of *Lm* while still retaining the ability to successfully deliver a eukaryotic plasmid for expression of tumor antigen (79). Recently, this concept of an *Lm*-based suicidal vector for tumor immunotherapy was advanced with the development of a new vector, *Listeria monocytogenes* recombinase-induced intracellular death (*Lm*-RIID). This strain features Aduro’s live-attenuated double deletion *Lm* vaccine (LADD) that has been further modified by the introduction of loxP sites adjacent to essential genes, as well as a gene for Cre recombinase that is inserted under the *actA* locus (78). In bacterial growth media, *Lm*-RIID proliferates and functions normally, however, once it infects host cells and gains entry to the cytosol, Cre-recombinase is produced and excises the essential genes flanked by loxP sites. The loss of these essential genes ultimately results in loss of viability for the bacterium, while still allowing sufficient time for it to produce and secrete the necessary tumor antigens within the cytosol. This construct has demonstrated efficacy similar to the LADD platform in mouse tumor models, inducing a robust anti-tumor immune response, and a substantial reduction in tumor burden. In combination with anti-PD-1 therapy, it conferred maximal protection against tumor growth and regression in a lung metastasis model. Perhaps the most interesting characteristic about this platform is its excellent safety profile. Although the enhanced attenuation of *Lm*-RIID did result in reduced immunogenicity in direct comparison to existing platforms, it was shown to be more immunogenic than the killed but metabolically active (KBMA) strain and similarly effective to the live, attenuated, double-deleted (LADD) platform (78). Of note, also, is the recombinant suicidal *Listeria monocytogenes* strain (*rsΔ2*) described by Sinha et al. (77) that undergoes autolysis upon entry into the cytosol but produces and delivers both protein antigen and a eukaryotic expression vector encoding the same antigen to an infected cell. This novel suicidal *Lm* vaccine induced both humoral and cell cytolytic responses to the model antigen, ovalbumin, when delivered both intramuscularly and orally. Importantly, delivery of only the eukaryotic expression plasmid encoding for the antigen by *rsΔ2* resulted in blunted responses compared to the vector that delivered *Lm*-produced protein antigen and the eukaryotic expression vector. These novel suicidal *Lm*-based vaccine vectors demonstrate that, while improvements may still be desired to match their anti-tumor efficacy to previously-developed live *Lm*-based vectors, their effectiveness when given orally, enhanced level of safety, and their greater level of versatility to deliver both protein and nucleic acid cargo demonstrates great promise for them as clinical vectors for tumor immunotherapy.

### *Listeria* as a Targeted Radionuclide Therapeutic (TRT) Platform

The combination of *Lm*-based immunotherapy with radiotherapy has shown promise as a synergistic treatment regimen in a preclinical model for melanoma (86). However, there are still challenges to discover the most efficient sequence of treatment administration for both the *Lm* vaccine and radiotherapy, i.e. concurrently or sequentially, and the off-target effects of radiotherapy on healthy tissue may complicate the timing and number of treatments (87, 88). To overcome these challenges, a recent study utilized a previously engineered *Lm*-based vaccine platform that specifically homes to and replicates within tumors as a vector for tumor-specific delivery of radiotherapy (29). Targeted radionuclide therapeutics (TRT) have shown potential in clinical trials as observed by various radionuclide/antibody conjugates that have been effective in treatment of malignancies such as glioblastoma (89) To accomplish this, a tumor-trophic *Lm*-based vector was incubated with anti-*Listeria* antibodies labeled with radionuclide, ^188^Re, thereby creating a tumor-targeting radiotherapy platform (30). This generated a radioactive attenuated *Lm* strain with high viability, stability, and infectivity. When this radioactive *Lm*-based therapy was administered in a murine pancreatic cancer model, radioactive^188^Re specifically accumulated in the tumors and metastatic lesions, further confirming the ability of *Lm* to act as a tumor-specific vector. Interestingly, while there was accumulation of this radioactive *Lm* in both kidney and liver, no histopathological damage and change in liver function was observed one week after the last treatment, suggesting that the highly proliferative tumor cells in metastases are more susceptible to the consequences of radiation-induced DNA damage than normal tissues (30).

However, a limitation of this strategy is the difficulty in generating this antibody dependent *Lm* radionuclide construct. This limitation was overcome in a more recent strategy that utilized radioactive phosphate ^32^P to generate a novel TRT *Lm* construct. This simple but elegant method involved starving the attenuated *Lm* in saline followed by culturing in media supplemented with ^32^P (29). The simplicity of this method allows for greater reproducibility and, more importantly, generation of an *Lm* construct that is more viable and stable without losing the incorporated radionuclide. This *Lm*-TRT also specifically homed to the tumor and metastatic sites in a pancreatic cancer model while demonstrating more effectiveness than its precursor. Interestingly, the major side effect associated with the use of ^32^P in cancer therapy, accumulation in the bone marrow, was not seen with *Lm*-^32^P used as a TRT. As this approach has only been assessed preclinically in a difficult-to-treat pancreatic cancer model, application of this technology to additional cancer models that are more amenable to immunotherapy may provide even more promising results.

### Incorporation of *Lm*-Derived Products Into Nanovaccine Platforms

As development continues in safer and more effective live *Lm*-based vaccines, other groups have focused on utilizing specific products or proteins from *Listeria monocytogenes* to improve the immunogenicity of nanoparticle-based vaccine platforms (90, 91). Much like *Lm*, nanoparticle-based vaccines, comprised of liposomes and/or metal particles, have the ability to selectively deliver cargo to professional antigen presenting cells, and accumulate within primary and metastatic tumors (92). In fact, a recently developed nanovaccine utilized the adjuvant properties of LLO to enhance the efficacy of a gold nanoparticle-based vaccine in preclinical development for metastatic melanoma (90). Gold nanoparticles are utilized in tumor nanovaccines due to their small size, ability to disseminate widely throughout the tissues, and ability to specifically accumulate at tumor sites (93). The gold nanoparticle-based vaccine, GNP-LLO_91-99_, was constructed by incorporating gold nanoparticles with β-D glucose, which increases tumor oxidative stress, and a H2k^d^-restricted epitope peptide derived from LLO, LLO_91-99_. GNP-LLO_91-99_ induced a robust production of inflammatory cytokines, a reduction in the intratumoral Treg and MDSC populations, and increased infiltration of LLO and tumor-specific CD8^+^ T cells into the tumor. Surprisingly, GNP-LLO_91-99_ was more effective at reducing melanoma tumor burden as a monotherapy, than ICI therapy. However, the combination of GNP-LLO_91-99_ with anti-PD-1 and anti-CTLA-4 antibodies resulted in synergistic efficacy and complete tumor regression (90) Of note, the melanoma model in this study, B16F10, is performed in the H2^b^-restricted C57BL/6 mice while the LLO-derived peptide within GNP-LLO_91-99_ is well characterized as an H2K^d^-restricted LLO epitope suggesting further investigation is warranted to fully understand its mechanism(s) of adjuvancy.

Another utilization of *Lm*-derived components in a nanovaccine involves the use of purified LLO in combination with liposomal nanoparticles (91). While liposomes are effective carriers of antigens for delivery to APCs (94), it was hypothesized that the lytic properties of LLO may allow for improved cytosolic release of liposomal cargo after uptake. The LLO-liposome nanoparticle-based vaccine consisted of a liposome, loaded with recombinant OVA protein, as a model tumor antigen, and recombinant LLO (91). When compared against a liposomal nanoparticle-based vaccine containing only ovalbumin, the incorporation of LLO into the nanovaccine resulted in enhanced presentation of the immunodominant CTL epitope, SIINFEKL, by APCs, improved OVA-specific cytolytic and humoral responses, a dramatic delay in growth of OVA-expressing melanoma tumors, and improved survival.

While the different types of nanovaccines have widely divergent compositions and chemistry, the incorporation of *Lm*-based components appears to dramatically improve their immunogenicity and anti-tumor efficacy (90, 91). These studies suggest that the utilization of *Lm* for tumor immunotherapy may not necessitate the need for the whole organism but just purified listerial components with unique properties to eventuate a safe but effective anti-tumor response.

### *Listeria* as a Gene Delivery Vector

Due to the cellular tropism of *Lm* and its ability to gain entry to the cytosol after infection, it is uniquely capable as a vector for gene delivery (84, 95, 96). In a process called bactofection, bacteria can be engineered to deliver eukaryotic expression vectors containing genes encoding for enzymes or protein antigens to an infected cell (97). In fact, previous studies have found *Listeria monocytogenes* to be a suitable vector for bactofection (84, 96, 98). Souders et al. performed the first demonstration that this ability of *Lm* to perform bactofection could be utilized in tumor immunotherapy (79). In this study, an *Lm*-based bactofection construct platform was made by engineering *Lm* to include a suicide cassette that expresses a phage lysin under the control of the *actA* promoter, and a eukaryotic expression system containing the HPV16 E7 tumor antigen. Upon infection of APCs, the *Lm* bactofection construction, *Lm*-V2, escaped into the cytosol and underwent autolysis while delivering the E7 expression vector. *Lm*-V2 allowed for proficient expression of the E7 tumor antigen and induced E7-specific CTLs that infiltrated E7-expressing tumors and delayed tumor growth. While *Lm*-V2 did not produce as robust an anti-tumor response as the *Lm*-LLO-E7 vector that secreted E7 protein in the cytosol and did not undergo autolysis, it served as a proof-of-concept for a strategy that would ensure safety as a primary feature (79). Schoen et al. also demonstrated the utility of *Lm* as a vector for bactofection of antigens in a study where, instead of a CMV-driven expression vector, they introduced mRNA encoding for OVA (99). Upon infection of APCs *in vitro*, this vector was able to facilitate the activation of OVA-specific CTLs. While no *in vivo* experiments were performed in this study, they also demonstrated the ability of *Lm* to deliver nucleic acids to tumor cells upon infection. This strategy of utilizing *Lm* as a vector for bactofection of tumor cells was furthered by work from Pijkeren et al. that described the development of an *Lm*-based bactofection vector engineered to release a eukaryotic expression plasmid encoding luciferase subsequent to antibiotic administration (100, 101). *In vitro* infection and intratumoral infection *in vivo* of this bactofection construct did result in robust luciferase expression after antibiotic treatment suggesting *Lm* may also find utility as a tumor-selective delivery vector for nucleic acids.

While *Lm* is certainly capable as a vector for gene therapy to deliver nucleic acid cargo and facilitate protein expression in a target cell, current constructs are still not as effective at delivering antigen than *Lm* constructs that encode and secrete protein directly (102). Thus, since this would limit the ability to maximally present antigens, the ability of these various constructs to elicit robust antigen-specific CD8^+^ and CD4^+^T cell responses is currently limited and this technology will likely require further development prior to its successful entry into clinical trials.

## Combination Therapies With *Lm*

*Lm*-based vaccines alone stimulate robust immune responses, increase immune infiltrates into tumors, and result in durable anti-tumor responses in many preclinical models of cancer (31). However, the heterogeneity of tumors and the TME in humans remains a major obstacle in obtaining effective responses to many cancer therapeutics in the broader population (103). One approach to overcome the detrimental effect of tumor heterogeneity on drug sensitivity is to utilize a combination therapy approach that consists of multiple drugs with complementary mechanisms of action (104). We describe in detail below combination therapy approaches utilizing *Lm*-based vaccines that have shown promise in treatment of cancers in the clinic and preclinically.

### *Lm* in Combination With Immune Checkpoint Inhibitors (ICIs)

One mechanism by which a tumor can escape immune-mediated destruction is by enhancing the expression of immune checkpoint molecules on T cells such as PD-1 and CTLA-4 (105). While effector T cells can transiently express these molecules during activation or prolonged activation, high levels of these molecules are associated with an exhausted T cell phenotype, thereby limiting their tumor lytic potential (106), Since the anti-tumor efficacy of *Lm*-based vaccines is through induction of potent tumor-specific CD8^+^ T cells, maintaining their functionality is necessary for an effective immunotherapeutic platform (3). In fact, blockading antibodies that act as immune checkpoint inhibitors (ICI) dramatically enhance the functionality of anti-tumor T cell responses, an attribute that has revolutionized the treatment of various solid tumors (105, 107). Therefore, combination of these agents with *Lm*-based immunotherapeutics, which are able to induce potent and effective CTLs that infiltrate the TME may lead to even greater survival in cancer patients.

Thus far, various preclinical studies have found a synergistic effect in the combination of *Lm-*based therapeutics with ICIs (108–111). The initial study demonstrating synergy between anti-PD-1 blockading antibodies and the anti-tumor efficacy of *Lm*-based vaccines was by Mkrtichyan et al. (110). This study utilized the well-characterized *Lm*-LLO-E7 vaccine and assessed the ability of ICI to improve anti-tumor responses against the TC-1 tumor model for HPV-associated cancers. While they did find a synergistic anti-tumor effect by combining *Lm*-LLO-E7 with anti-PD-1 antibody, the dosage of *Lm*-LLO-E7 was approximately 5-fold lower and less effective than previous studies which may have better revealed the synergistic effect of these two therapeutics (110). Further support for this finding was found in a study assessing the efficacy of an *Lm*-based vaccine in combination with PD-1 blockade in a preclinical model of pancreatic ductal adenocarcinoma (PDAC) (109). PDAC is a highly heterogeneous solid tumor that is categorized as a “cold” tumor with limited immune cell tumor infiltrates making it less receptive to ICI treatment alone. However, utilizing an *Lm*-based construct expressing Annexin A2 (*Lm*-ANXA2), a relevant PDAC tumor antigen, Kim et al., demonstrated that sequential treatment with *Lm*-ANXA2 followed by PD-1 blockade resulted in an increase in overall survival of PDAC-bearing mice compared with either *Lm*-ANXA2 or anti-PD-1 therapy alone. Moreover, this therapeutic strategy elicited strong ANXA2-specific immune responses in the TME as evidenced by increase production of IFNγ, an observation not found in the group receiving anti-PD-1 alone (109) In addition to PDAC and HPV-associated cancers, *Lm*-based vaccines have also been reported to synergize with ICI in a particularly challenging malignancy with low survival rates, hepatocellular carcinoma (HCC). In fact, unlike many other cancers, ICI has limited efficacy and can even lead to hyperprogression in some patients (112, 113). Interestingly, an *Lm*-based vaccine expressing a multivalent HCC tumor antigen, Lmdd-MPFG, can induce PD-L1 expression, the ligand for PD-1, in HCC tumor cells suggesting that it may synergize with ICI therapy (108). When Lmdd-MPFG was administered along with anti-PD-1 antibody, it resulted in significantly reduced tumor burden as compared to PD-1 blockade or *Lm* vaccine treatment alone. While Lmdd-MPFG alone did result in significant anti-tumor efficacy, PD-1 blockade alone did not, mirroring clinical experience, and suggesting that the *Lm*-based vaccine sensitized HCC to ICI. Further evidence suggested that the ability of Lmdd-MPFG to polarize TAMs to the M1 phenotype played a role in the sensitization of HCC to PD-1 blockade (108). In addition to ICI synergizing with *Lm*-based vaccines in a therapeutic setting, it has also been found to enhance protection against tumor challenge in an murine model of melanoma expressing ovalbumin, B16F10-OVA (111). While administration of the *Lm*-based vaccine expressing OVA_257-264_ fused to ActAN100, *Lm*-OVA, provided significant protection against subsequent challenge with B16F10-OVA, up to 30% of mice developed tumors. The authors hypothesized that this could be due to the induction of peripheral tolerance mechanisms such as immune checkpoints that allow tumor escape. Therefore, approximately 2 months after *Lm*-based vaccination, mice were challenged with tumor, and received ICI (either anti-PD-1, anti-CTLA-4, or anti-PD-L1) every 10 days subsequently, in order to maintain anti-tumor T cell function. Each of the ICI antibodies enhanced the anti-tumor effect of *Lm-*OVA. More strikingly, there was significantly more mice remaining tumor-free after the addition of checkpoint blockade compared to vaccination with *Lm*-OVA alone. The data from these studies suggest that the efficacy of *Lm* vaccines can be enhanced by ICI therapy but *Lm* vaccination can also sensitize previously “cold” tumors, such as PDAC, to the powerful potential of ICI therapy (108, 111).

Use of checkpoint inhibitors in combination with *Lm* therapy is also being currently evaluated in various phases of clinical trials (**Table 1**). As we discussed in section 1.3.2, a Phase II study has demonstrated the positive immunomodulatory effect of the combination therapy of CRS-207 (*Lm*-mesothelin) with nivolumab (anti-PD-1) on the TME in the treatment of human PDAC (56).

### *Lm* Combination With Adoptive Cell Therapy (ReACT Cells)

Adoptive cell therapy (ACT) is the use of engineered or naturally occurring immune cells for the treatment of cancer (114). This strategy has enjoyed unprecedented success in oncotherapy, with high response rates in hematopoietic malignancies and melanoma (114). However, this strategy comes with serious limitations and drawbacks that make its use limited in the therapy of solid tumors (115). One of the most significant challenges is that the TME is an immunosuppressive environment with elevated levels of anti-inflammatory cytokines, inhibitory receptor ligands, and immunosuppressive cell types such as MDSCs that impede the activation, proliferation and differentiation of anti-tumor immune cells (116). Due to their ability to modulate the TME and reduce peripheral tolerance mechanisms, *Lm*-based vaccines may be uniquely able to overcome the limitations of this therapeutic strategy and enhance the efficacy of ACT (50). While *Lm* improves the ability of immune cells to infiltrate into the TME, *Lm* is also able to infect MDSCs which specifically home to the TME and likely mitigate immunosuppression within the tumor (117). Xin et al., has developed an approach that utilizes *Lm*-based vaccines to enhance ACT and overcome the immunosuppressive TME in a strategy named Reenergized ACT (ReACT). To advance their work, they utilized mono-specific and dual-specific CD8^+^ T cells that recognize a tumor antigen, gp100, or both a tumor antigen and an *Lm*-derived antigen, OVA, respectively. After allowing for tumors to establish, mice were administered mono or dual-specific CD8^+^ T cells followed by intratumoral injection of the *Lm*-OVA vaccine. Surprisingly, mice receiving only CTLs did not respond and less than 10% of mice receiving only tumor-specific CTLs and i.t. *Lm*-OVA responded, In contrast, 69% of mice receiving both i.t. *Lm*-OVA injection and dual-specific CTLs that recognize the tumor and *Lm*-derived antigens responded and eradicated their tumors. This method also resulted in greater tumor infiltration and function of the transferred CTLs and reduced expression of immune checkpoint molecules by CTLs. In the TME, MDSCs and Tregs made up a lower percentage of overall tumor cells in each of the *Lm*-treated groups but only the dual-specific CTLs with *Lm* treatment group had a higher Teff/Treg ratio and lower absolute Tregs than other treatment groups (14, 15). As loss of the targeted antigen is associated with therapeutic resistance in ACT, broadening of the anti-tumor response through epitope spreading to additional tumor antigens should result in a more durable anti-tumor response (118, 119). In fact, this therapeutic strategy did lead to epitope spreading that induced long-lasting endogenous memory cells that provided protection against subsequent challenge (14). In this preclinical model of melanoma, the only adverse events associated with ReACT was limited to mild vitiligo at the primary tumor injection site (14).

### *Lm* in Heterologous Vaccination Schedules

Heterologous prime boost involves the administration of the same antigens using different vaccine vector platforms to generate a more robust immune response than a homologous prime boost vaccine regimen (120, 121). *Lm*-based vaccines are particularly effective at generating robust responses when used as a booster or primer in combination with various vaccine vectors including viruses, DNA and peptides (122–125). In a preclinical study that involved the use of a DNA vaccine encoding the prostate-specific antigen prostatic acid phosphatase (PAP) along with an attenuated *Lm* vaccine, LADD-PAP, greater antitumor efficacy was seen in the prime boost regimen as compared to the DNA vaccine only regimen or the LADD-PAP only regimen (122). This heterologous vaccination schedule interestingly led to the induction of more CD4^+^ T cells, which may have played a role in the enhanced antitumor immune response. Enhanced cellular immune responses were observed with a heterologous prime boost regimen consisting of an EGFRvIII peptide vaccine, PEPvIII, and an *Lm*-based vaccine targeting EGFRvIII, *Lm*-EGFRvIIIx5, which resulted in greater induction of EGFR-specific CD8^+^ T cells than either vaccine alone (125). Interestingly, this enhanced EGFR-specific CTL response was only observed when the *Lm*-EGFRvIIIx5 was given as a booster to a previous peptide immunization. *Lm*-based vaccines have also been found to boost anti-tumor immunity when delivered in heterologous prime boost regimens with viral vectors (123, 124). In a prophylactic study, mice receiving a modified vaccinia Ankara vaccine expressing human p53, MVA-p53, followed by an *Lm*-based vaccine expressing human p53, *LmddA*-LLO-p53, resulted in greater anti-tumor protection against the murine breast cancer model, 4T1p53 (124). Further, the use of an *Lm*-based vaccine targeting the model tumor antigen chicken ovalbumin, *Lm*-OVA, prior to boosting with an oncolytic Maraba virus, MRB-OVA, resulted in a greater reduction in B16F10-OVA melanoma tumor growth, more tumor-free mice, and greater overall survival than mice receiving an adenovirus-based vaccine as the priming agent (123). Most importantly, this incorporation of *Lm*-based vaccines into heterologous prime boost approaches for cancer immunotherapy has already been found effective in a clinical trial. As we previously discussed, in a Phase II trial in PDAC patients, CRS-207 (*Lm-*mesothelin) in combination with Cy/GVAX improved median overall survival and enhanced induction of mesothelin specific CD8^+^ T cells as compared to Cy/GVAX alone (53).

### *Lm* in Combination With Radiotherapy

Radiation therapy (RT) has been a mainstay therapeutic option for nearly a century in the treatment of various forms of malignancies (126). However, recent work has found that RT does not solely disrupt tumor cell division but has immunostimulatory effects in the TME (127). RT helps trigger the release of tumor antigens, improve presentation of tumor antigens to T cells, and induces an inflammatory response in the TME that results in elevated IFN**γ** levels and reduced immunosuppression (86, 128). However, despite all these attractive features of RT, resistance and recurrence still occur through multiple mechanisms (129). In order to improve the efficacy of RT, a recent study utilized a combination of RT and *Lm*-OVA in the B16-OVA melanoma model (86). This approach resulted in an increase in the activated T cells infiltrating the TME and a more robust response in the combination therapy than in the use of *Lm* or RT alone. One possible explanation for the observed synergy was that both the *Lm* vaccine and the RT seem to activate different components of the immune system (86). A similar synergistic effect of RT and *Lm*-based vaccines was observed in a murine model of prostate cancer. In this study, an *Lm*-based vaccine expressing human prostate antigen (PSA) in combination with RT resulted in synergistic induction of PSA-specific splenic T cell responses and therapeutic anti-tumor efficacy (130).

In an effort to improve on the efficacy and specific delivery of the lethal radiation therapy to the tumors and metastasis, the Gravekamp group has developed an elegant method to use *Lm* as a delivery vector for radioisotopes directly to tumors. In this study using *Lm* infused with ^32^Phosphorous (L*m*-^32^P), this group was able to demonstrate that *Lm*-^32^P specifically homes to the TME in a mouse model for PDAC (29). The DNA damage caused by the radioisotope coupled with the antitumor efficacy of *Lm* itself makes this platform very potent and promising in cancer therapy.

### *Lm* in Combination With Inhibitors of Complement Signaling

The complement cascade is well characterized to play a crucial role in the innate immune response to pathogens (131). However, evidence over the past decade demonstrates that the complement pathway and its components can also regulate adaptive immunity, particularly anti-tumor T cell responses (132). In fact, complement is highly activated in tumors and this activation results in T cell dysfunction and blunted anti-tumor responses (133). Importantly, a small molecule inhibitor of the C5a receptor (C5aR1), PMX53, enhances infiltration and function of tumor-infiltrating T cells leading to tumor regression in mouse cancer models (134). Therefore, inhibition of complement signaling may make the TME more amenable to anti-tumor T cells and synergize with an *Lm*-based vaccine. In a recent study, this synergistic potential was confirmed by demonstrating that treatment with PMX53 synergized with tumor vasculature targeting *Lm*-based vaccines to significantly reduce primary tumor growth and reduce lung metastases in a murine model of metastatic breast cancer, 4T1 (135). Furthermore, this synergistic potential was correlated with reduced levels of MDSCs and Tregs in the lungs of tumor-bearing mice and enhanced maturation of lung-associated dendritic cells.

### *Lm* in Combination With Traditional Chemotherapy

Chemotherapy remains the gold standard for the treatment of various forms of cancers. Chemotherapy may also synergize with and augment the activity of immunotherapies, such as *Lm*-based vaccines, by inducing direct cell death that results in release and presentation of tumor antigens and maturation of dendritic cells (136, 137). Chemotherapy, such as low-dose cyclophosphamide, can also significantly reduce the Treg population in the TME (138). In fact, in a murine model of hepatic metastases, animals treated with attenuated *Lm* along with low dose cyclophosphamide had reduced levels of Tregs in the TME and spleen and a dramatic increase in overall survival (139). The Treg-depleting properties of cyclophosphamide were also found to enhance the efficacy of an *Lm*-based vaccine in the challenging KPC mouse model for PDAC (140, 141). In this study, Treg depletion by cyclophosphamide was further enhanced by addition of anti-CD25 depleting antibody in combination with an *Lm-*based vaccine targeting a 25 amino acid region of Kras^G12D^, LM-Kras. In KPC mice less than 2 months old, the combination of LM-Kras and cyclophosphamide/anti-CD25 was found to significantly delay progression of precancerous pancreatic lesions to PDAC and enhance survival in comparison to LM-Kras or cyclophosphamide/anti-CD25 alone. T cell infiltration and Th1 responses in the pancreas were also enhanced in the group receiving LM-Kras and cyclophosphamide/anti-CD25 in comparison to LM-Kras alone. While it is not possible to dissect the exact contribution of cyclophosphamide in the efficacy of LM-Kras in this study, much like another PDAC study that included cyclophosphamide in all treatment groups (109). These results provide additional pre-clinical evidence that chemotherapy will likely have an important role to play in effective treatment regimens with *Lm*-based vaccines. In humans, several clinical trials have incorporated chemotherapeutic agents, such as cyclophosphamide, pemetrexed and cisplatin, along with *Lm*-based vaccines into their treatment regimen (41, 44, 53, 55, 56, 60). As yet, evidence from these clinical trials suggest some chemotherapy regimens may provide a clinical benefit in combination with *Lm*-based vaccines (53, 60) while others have found mixed or negligible benefit from this combination (41).

### Conclusion

The recent results reported from several clinical trials demonstrate the promising future of *Lm*-based tumor immunotherapies but also reveal challenges that educate the future development of the platform (41, 42, 60). Numerous improvements to the platform have already been reported in preclinical studies that would not be evident in the clinical results as they are mostly utilizing first-generation *Lm*-based constructs (29, 30, 78). Even with the use of these first generation constructs, promising clinical responses have been reported in several cancers including cervical cancer, malignant pleural mesothelioma, and canine osteosarcoma (74). As results from clinical and preclinical studies demonstrate, improvements to therapeutic efficacy may be realized utilizing *Lm*-based vaccines in heterologous prime boost regimens with other vectors and by combination with synergistic therapeutic strategies such as ACT and ICI (14, 53, 108, 110, 122, 123, 134). However, there still remain several challenges going forward to realize the full potential of Lm-based vaccines. As a recent clinical trial suggests, *Lm-*based vaccines may not significantly benefit from combination with particular chemotherapies that suppress immunity and increase risk of adverse events (41). Further, while rarely observed in clinical trials as yet, the incidence of listeriosis in humans and dogs may justify additional study into the variables contributing to this risk (43, 76). Nevertheless, improvements to clinical safety may be realized with the adoption of recently developed suicide strains, such as *Lm*-RIID, that would dramatically reduce the risk of listeriosis (78). As with other current vaccine platforms, treatment resistance due to immune escape will also likely be an ongoing challenge, particularly due to many of the current vaccines targeting a single tumor antigen (142, 143). While not yet published, improvements to the antigens targeted by *Lm*-based vaccines such as the multivalent ADXS-HOT platform targeting immunogenic hotspot mutations and the patient-personalized ADXS-NEO platform will likely bring improvements in efficacy, in part by limiting immune escape. Finally, recent advancements in leveraging its tumor-trophic potential suggest that attenuated *Lm*-based therapeutics can provide multiple separate but effective anti-tumor mechanisms which, if fully leveraged, may also mitigate therapeutic resistance (14, 15, 29, 30, 117, 144). With our improved understanding of its clinical performance and the continued development of the platform, the future is promising for *Lm*-based therapeutics.

## Author Contributions

MO, drafting. YP, revision. LW, drafting and revision. All authors contributed to the article and approved the submitted version.

## Funding

National Institutes of Health 1R15CA216205-01 (LW).

## Conflict of Interest

YP has a financial interest in Advaxis, Inc., a vaccine and therapeutic company that has licensed or has an option to license all patents from the University of Pennsylvania that concern the use of *Listeria monocytogenes* or listerial products as vaccines.

The remaining authors declare that the research was conducted in the absence of any commercial or financial relationships that could be construed as a potential conflict of interest.
